# Carbon Nanotube Integration with a CMOS Process

**DOI:** 10.3390/s100403857

**Published:** 2010-04-15

**Authors:** Maximiliano S. Perez, Betiana Lerner, Daniel E. Resasco, Pablo D. Pareja Obregon, Pedro M. Julian, Pablo S. Mandolesi, Fabian A. Buffa, Alfredo Boselli, Alberto Lamagna

**Affiliations:** 1 Grupo MEMS, Comision Nacional de Energia Atomica, San Martin 1650, Buenos Aires, Argentina; E-Mails: mperez@cnea.gov.ar (M.S.P.); boselli@cnea.gov.ar (A.B.); alamagna@cnea.gov.ar (A.L.); 2 School of Chemical, Biological and Materials Engineering, University of Oklahoma, Norman, OK 73019, USA; E-Mail: resasco@ou.edu; 3 Departamento de Ingenieria Electrica y de Computadoras, Universidad Nacional del Sur, Bahia Blanca B8000FTN, Argentina; E-Mails: pablopareja@ieee.org (P.D.P.O.); pjulian@uns.edu.ar (P.M.J.); pmandolesi@uns.edu.ar (P.S.M.); 4 INTEMA Facultad de Ingenieria, Universidad Nacional de Mar del Plata, Mar del Plata B7608FDQ, Argentina; E-Mail: fbuffa@fi.mdp.edu.ar

**Keywords:** carbon nanotube sensor, CMOS integration, microchip sensor, SWCNT, PACS 85.35.-p, 85.35.Kt, 85.40.-e

## Abstract

This work shows the integration of a sensor based on carbon nanotubes using CMOS technology. A chip sensor (CS) was designed and manufactured using a 0.30 μm CMOS process, leaving a free window on the passivation layer that allowed the deposition of SWCNTs over the electrodes. We successfully investigated with the CS the effect of humidity and temperature on the electrical transport properties of SWCNTs. The possibility of a large scale integration of SWCNTs with CMOS process opens a new route in the design of more efficient, low cost sensors with high reproducibility in their manufacture.

## 1. Introduction

The development of carbon nanotube (CNT) sensors has been the subject of intense research in recent years. Due to their unique physical and electrical proprieties, CNT sensors have been shown to be good sensing elements for pressure [1], alcohol [2], gases [3,4] and biological molecules [5,6].

CNT sensors are mostly manufactured using basic lithography processes [7,8], in which reproducibility and resolution are limited to the manufacturing laboratory, making it difficult to scale up to volume manufacturing. Here we use complementary metal-oxide semiconductor (CMOS) technology to manufacture single wall carbon nanotube (SWCNT) sensors. This technology has been used for several years by the semiconductor industry, obtaining excellent reproducibility results in manufacturing. The CS are manufactured in batch technologies, where thousands of these can be generated from a single wafer, hence the cost of manufacturing is extremely reduced. In addition, this technology allows higher sensitivity through on chip signal processing.

To place the SWCNTs on the electrodes, a dielectrophoretic (DEP) process is used to improve the efficiency of SWCNT sensor fabrication. DEP is a phenomenon where neutral particles undergo mechanical motion inside an AC electric field [9,10]. The DEP force is a simple and effective methodology to assemble SWCNTs on electrodes and is compatible with commercial CMOS process [11].

As a result, these sensors are the union of sensing elements, interfacing and measurement circuitry in a single integrated chip with low power consumption, high reproducible manufacture and low cost, making possible the scale up of the sensors from the research laboratory prototype to a commercial product.

We investigated with the CS the effect of humidity and temperature on the electrical transport properties of SWCNTs. The experimentally transport properties of SWCNT network as a function of temperature are found consistent with a fluctuation induced tunneling mechanism due to the existence of contact barriers between individual nanotubes [12]. The energy barriers can exist as a result of contacts either between metallic and semiconductive nanotubes or between semiconductive nanotubes with different band gap.

## 2. Experimental Section

### 2.1. Carbon Nanotubes

**Figure 1.**
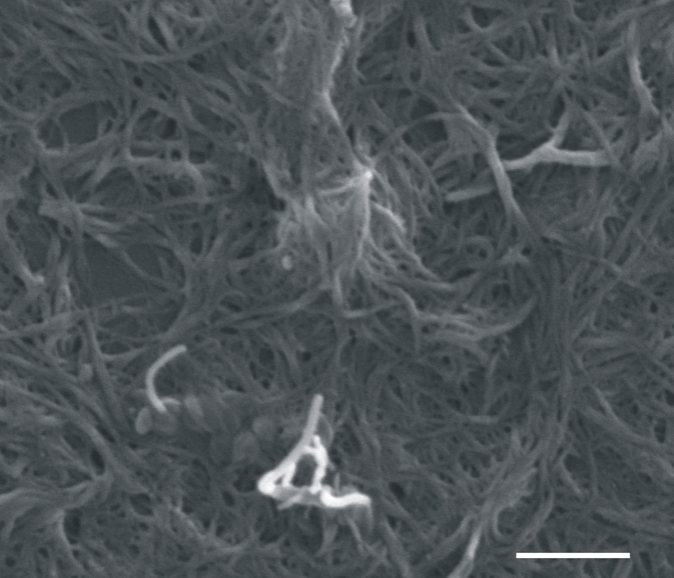
SEM micrography of SWCNTs. Scale bar: 100 nm.

The carbon nanotubes used in this study have been obtained by the catalytic CoMoCAT method [13], which employs a silica-supported Co-Mo powder to catalyze the selective growth of SWCNTs by disproportionation of CO. The SWCNTs grown by this method were purified by SWeNT (Southwest Nanotechnologies). The resulting nanotubes have an excellent quality [14] as determined by transmission electron microscopy (TEM), scanning electron microscopy (SEM) (Figure 1) and the D/G band ratio in the Raman spectra obtained at laser excitations of 633, 514, and 488 nm, as well as very low impurity content as determined by XPS analysis. The SWCNT used in this experiment have a semiconduting character [15,16] and have an average length of 300 nm.

### 2.2. Chip Sensor

The top layer metal in the CMOS chip was designed to act as electrodes for the CS fabrication. Openings of the passivation layer were made to expose the electrodes in the CS design.

**Figure 2.**
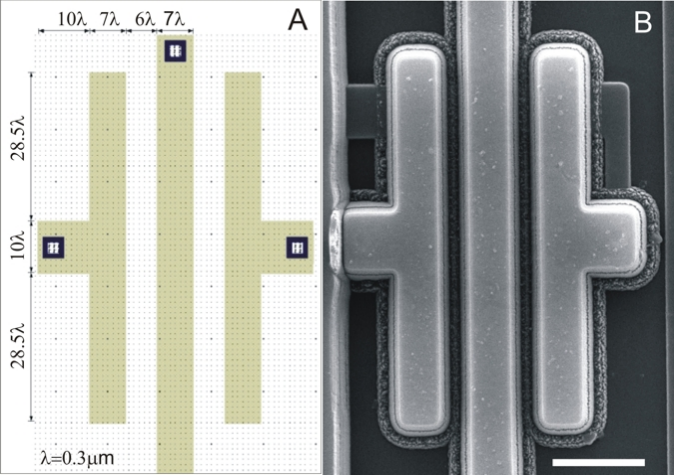
(A) Mask layout with electrode dimensions, (B) SEM micrography of the electrodes. The gap between the electrodes is 1.3 μm. Scale bar: 10 μm.

The CS was fabricated using a commercial CMOS process; this achieves the required resolution with low cost and high reproducibility. The interface between the SWCNTs and the output signal was composed of a microelectronic circuit. This circuit takes the signals inherent to the measurement, and generates the amplification needed to obtain an output signal with low noise level. The amplification was made through a transresistance amplifier circuit. The integrated circuit was fabricated by MOSIS in the standard process AMIS 0.50 with a minimum size *λ* = 0.3 μm. The design was made using Tanner L-Edit software. Figure 2 shows the dimensions of the electrodes.

### 2.3. Carbon Nanotube Deposition

**Figure 3.**
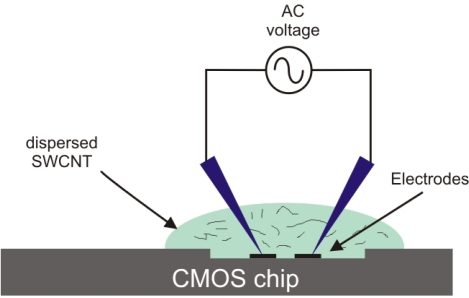
Scheme of the SWCNTs deposition by DEP process.

**Figure 4.**
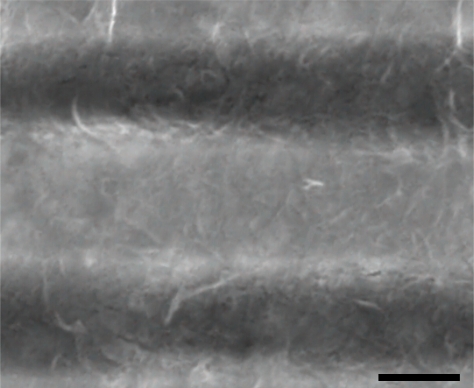
SEM micrography of SWCNTs deposited between electrodes. Scale bar: 1 μm.

**Figure 5.**
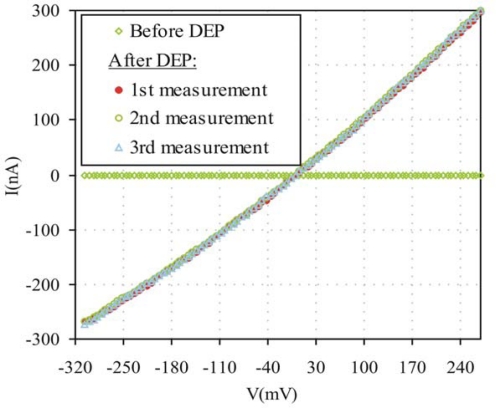
Current voltage characteristics of the SWCNTs assembled onto CMOS circuitry by DEP process.

We immersed the chip in 50% nitric acid solution for 12 s to remove the aluminum oxide layer formed on top of electrodes. Immediately after that, SWCNTs deposition was made over the chip. We used the DEP process to deposit the SWCNTs on the electrodes. An amount of 0.2 mg of SWCNTs were dispersed in 1 mL ethanol and ultrasonicated for 20 min with a high power horn sonicator. One microliter of this solution was put between the gap of the electrodes and an alternating voltage of 5 Vpp at a frequency of 1 MHz was applied to the opposite drain electrodes to generate the DEP force (Figure 3). After a few minutes, the ethanol was evaporated and the SWCNTs were aligned between the electrodes. Figure 4 shows the SWCNTs deposited between electrodes and Figure 5 shows stable I–V measurements before and after assemble SWCNTs, indicating that the DEP process worked correctly.

### 2.4. Humidity Control and Electrical Measurements

CS were located in a chamber (volume 500 cc) where air flow at a constant rate of 300 sccm and humidity was controlled with a 0.1% precision at 298 K. Electrical measurements were taken immediately after the SWCNT deposition in order to avoid interference by aluminum oxide formation over the electrodes. Current was supplied by a Keithley 6221 current source and voltage changes were measured with a Keithley 2000 multimeter. Programming to manage data acquisition was performed in LABVIEW (National Instruments).

## 3. Results and Discussion

### 3.1. Chip Sensor Layout

**Figure 6.**
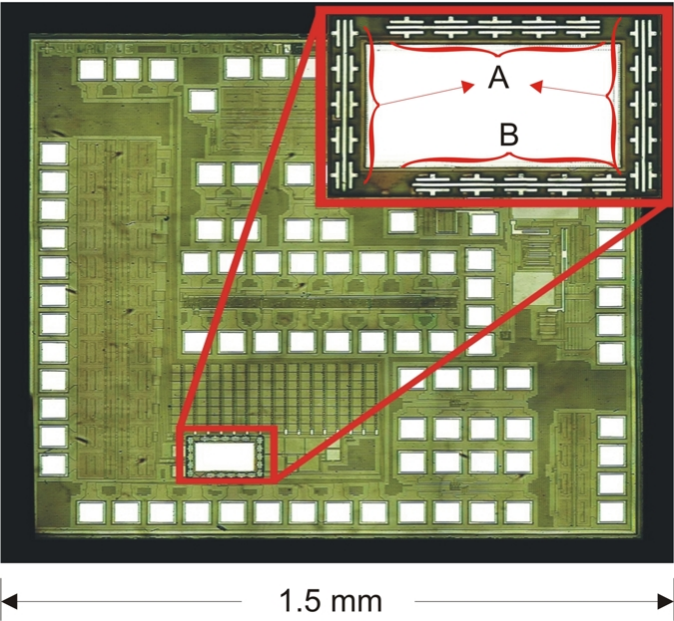
Optical microscopy of the full chip. Inset shows the opening in the passivation layer with exposed electrodes. “A” indicates the electrodes connected to the amplifier. “B” indicates the free electrodes connected directly to the external pads.

**Figure 7.**
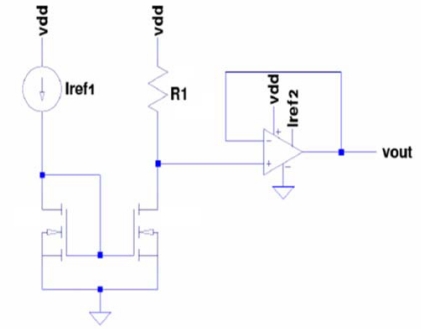
Schematic of the amplifier. Vdd: 5V, current reference 1 (Iref1): 2 μA, current reference 2 (Iref2): 10 μA, R1: SWCNT resistance; Vout: output voltage.

We designed the CS with a free window on the passivation layer that allowed the deposition of SWCNTs over the electrodes. A scheme of the CS is shown in Figure 6. The CS has an arrangement of free electrodes formed by one common central source and several drains connected directly with the external pads and one arrangement of electrodes connected to an amplifier. The amplifier circuit comprises a current mirror, which reflects the input current of one of the pads on the SWCNTs to generate an output voltage (V_out_) (Figure 7). This V_out_ is the input of an operational amplifier in a unity gain configuration, whose purpose is to act as a buffer and prevent the output voltage of the SWCNTs to be loaded or affected by any external circuit to the die.

### 3.2. Effect of Humidity on the Electrical Transport Properties of SWCNTs

**Figure 8.**
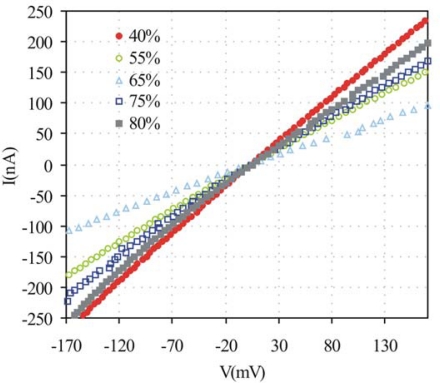
The effect of humidity over current voltage characteristics of SWCNTs detected through the free electrodes.

We examined the effect of the humidity changes on the resistance of SWCNTs. Figure 8 shows the effect of humidity over the current voltage characteristics of SWCNTs placed on the free electrodes. Under a modest humidity, the adsorbed water molecules appear to compensate the hole carriers in the SWCNTs, resulting in a increase in the resistance. When the humidity level becomes higher than 67%, the decreasing resistance is probably due to a surplus electron carrier. The possible influence of a contact resistance between electrodes and SWCNTs was recently addressed by other authors [17–19] and ruled out because the contact resistance present in two leads contacted samples provide a comparatively low contact resistance relative to the resistance between the electrodes and the possible Schottky barriers formed at the SWCNTs-electrode interface have a negligible effect on the measurements.

**Figure 9.**
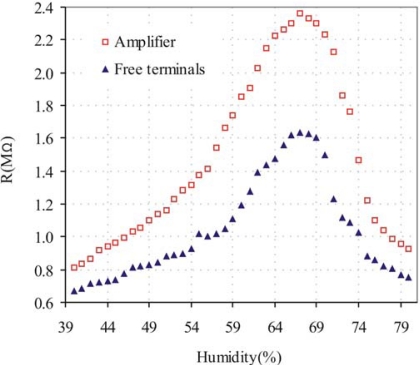
Effect of humidity over the resistance of SWCNTs detected by electrodes connected to the amplifier and free electrodes.

We have also compared the effect of humidity on the SWCNTs placed on the electrodes connected to the amplifier with the ones placed on free electrodes. Figure 9 shows the simulated resistance of the SWCNTs made with Spice software from V_out_ values using MOSIS parameters, compared with the SWCNTs resistance on the free electrodes. SWCNTs behavior as function of humidity changes was similar between electrodes connected to the amplifier and the free electrodes, and it is in agreement with what was previously reported by other authors [20,21], indicating that the CS works properly and the integration of CMOS process with SWCNTs was successful.

A difference (approx. 0.2–0.8 Mohm) between the resistance with the amplifier and the free electrodes was observed with a same humidity value. This difference can be attributed to a different amount of SWCNTs deposited between each electrode.

The resistance values taken by the electrodes connected to the amplifier showed more uniformity than those taken by the free electrodes, demonstrating that the electrodes connected to amplify have a better output signal with low noise level.

### 3.3. Effect of Temperature on the Electrical Transport Properties of SWCNTs

We studied the electrical transport characteristics of SWCNTs with the CS at different temperatures. Among the theoretical models that have been proposed to explain the observed experimental features in disordered heterogeneous systems [22,23] the Fluctuation Induced Tunneling (FIT) model [24] has been subject of attention [25]. The conduction electrons are delocalized here and free to move over very large distances as compared to atomic dimensions. In these systems electron transfer between large conductive segments separated by small insulating gaps dominates electrical conductivity.

**Figure 10.**
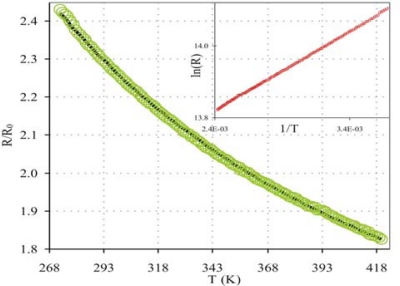
Resistance *vs*. temperature for SWCNTs deposited on electrodes connected to the amplifier. Line is fit to the data obtained from Equation (1) with fit parameters: T_1_ = 277 K and T_0_ = 40 K. The inset shows the data in a Ln(R) *vs.* 1/T plot.

The R (T) dependence of SWCNT network was measured in the range of 273 to 420 K (Figure 10). In this temperature range, R (T) dependence can be approximated by a FIT conductivity mechanism [12,24]:

(1) $$R=R_{0}e^{T_{1}/(T+T_{0})}$$

In this expression R_0_ is the resistance at room temperature, $T_{1}=2{\mathrm{SV}}_{0}^{2}/\left(\pi k_{B}e^{2}w\right)$ and $T_{0}=4\hbar {\mathrm{SV}}_{0}^{3/2}/(\pi^{2}w^{2}k_{B}e^{2}\sqrt{2m})$ with *S* and *w* being the junction surface and width respectively, V_0_ is the depth of the potential well, *m* the electron mass, *e* the electron charge, and k_B_ and *h* (*ħ* = *h* / 2*π*) are the Boltzmann and Planck constants, respectively. The solid curve in Figure 10 fits to the data based on Equation (1). The fit is very good over the whole temperature range studied suggesting, in accordance with was previously reported by other authors [19,26], that FIT mechanism can be used for these aligned SWCNTs on CS.

Here, it was shown that SWCNTs have a strong dependence of electrical resistance with temperature, which suggests their potential use of the CS as a thermistor.

## 4. Conclusions

In this work the feasibility of integration of a CMOS process with SWCNTs has been demonstrated. A dielectrophoresis assembly was used to deposit the SWCNTs over the electrodes. We successfully developed and tested at different humidities and temperatures a CS prototype which includes an amplifier and free electrodes. The SWCNT’s resistance was shown to be sensitive to humidity changes within a specific range. Although the charge transfer effect from adsorbed H_2_O molecules tends to saturate at a certain point, care for the humidity condition must be taken for SWCNT sensor applications. The charge carrier properties in SWCNTs between electrodes were studied at different temperatures. Experimental data was consistent with the fluctuations induced tunneling model that emphasizes the role of energy barriers between the nanotubes. CMOS technology has been used for several years in electronic devices. In this work we propose to develop a new application of this technology through which sensors with low power consumption, excellent precision and low cost can be obtained.
